# Frailty Predicts Poor Prognosis of Patients After Percutaneous Coronary Intervention: A Meta-Analysis of Cohort Studies

**DOI:** 10.3389/fmed.2021.696153

**Published:** 2021-08-19

**Authors:** Peng Wang, Shutang Zhang, Ke Zhang, Jie Tian

**Affiliations:** Department of Gerontology, Fuling Central Hospital of Chongqing City, Chongqing, China

**Keywords:** frailty, percutaneous coronary intervention, mortality, major adverse cardiovascular events, meta-analysis

## Abstract

**Background:** Frailty has been related to a higher risk of cardiovascular events, while the association between frailty and outcomes for patients with coronary artery disease (CAD) after percutaneous coronary intervention (PCI) remains unclear. We performed a meta-analysis of cohort studies to evaluate the above association.

**Methods:** Cohort studies aiming to determine the potential independent association between frailty and clinical outcomes after PCI were identified by search of PubMed, Embase, and Web of Science databases from inception to February 22, 2021. A random-effects model that incorporates the possible heterogeneity among the included studies was used to combine the results.

**Results:** Ten cohort studies with 7,449,001 patients were included. Pooled results showed that frailty was independently associated with higher incidence of all-cause mortality [adjusted risk ratio (RR) = 2.94, 95% confidence intervals (CI): 1.90–4.56, *I*^2^ = 56%, *P* < 0.001] and major adverse cardiovascular events [(MACEs), adjusted RR = 2.11, 95% CI: 1.32–3.66, *I*^2^ = 0%, *P* = 0.002]. Sensitivity analyses limited to studies including elderly patients showed consistent results (mortality: RR = 2.27, 95% CI: 1.51–3.41, *I*^2^ = 23%, *P* < 0.001; MACEs: RR = 2.44, 95% CI: 1.44–4.31, *I*^2^ = 0%, *P* = 0.001). Subgroup analyses showed that characteristics of study design, follow-up duration, or type of PCI did not seem to significantly affect the associations (*P*-values for subgroup analyses all >0.05).

**Conclusions:** Frailty may be an independent risk factor of poor prognosis for patients with CAD after PCI.

## Background

Currently, coronary artery disease (CAD) remains one of the most important causes of morbidity and mortality for global population, particularly for the elderly (1). Besides optimized medical treatment, early coronary revascularization has been established as the most effective therapy for alleviating symptoms and improving prognosis in patients with CAD (2). Due to the efficacy and invasiveness of the procedure, percutaneous coronary intervention (PCI) has become the most widely used method for coronary revascularization (3). For patients with acute CAD, such as ST-segment elevation myocardial infarction (STEMI), primary PCI is recommended as early as possible to avoid the necrosis of myocardium (4). For patients with stable CAD and frequent symptom of angina, elective PCI is also recommended to restore the coronary blood flow for the ischemic myocardium (5). With the development of the devices and techniques, increasing elderly patients with CAD received PCI (6). According to previous studies, more than 20% of patients that received PCI are older than 75 years (3, 6). However, despite of the overall effectiveness of the procedure, adverse cardiovascular events or event deaths remain occur in some patients after PCI, which highlights the importance of risk stratification for CAD patients that received PCI (7).

Frailty is a geriatric syndrome characterized by age-related decrease of reserve capacity of various systems and lack of resilience to stressors (8). Accumulating evidence suggests that frailty is related to poor prognosis of patients with various cardiovascular conditions, such as acute myocardial infarction (AMI) (9), congestive heart failure (10, 11), atrial fibrillation (12), and for patients after transcatheter aortic valve implantation (13). However, the association between frailty and the prognosis of patients after PCI remains unclear (14). Most studies showed that frailty is independently associated with higher risk of mortality and adverse events after PCI (15–22), while some did not (23, 24). Accordingly, a previous meta-analysis included eight cohort studies and showed that frailty was associated with a higher risk of death for patients after PCI (25). However, two of the cohort studies actually included patients who did not receive PCI (26, 27). Besides, this meta-analysis included studies with univariate analysis and a study using continuous gait speed as the indicator of frailty (28), which made the results of the meta-analysis difficult to interpret. Since several relevant cohort studies (17–22, 24) have been published since the previous meta-analysis, we aimed to perform an updated meta-analysis to summarize the current understanding for the association between frailty and prognosis after PCI.

## Methods

The Meta-analysis of Observational Studies in Epidemiology (MOOSE) guideline (29) and Cochrane's Handbook (30) was followed in this study.

### Literature Search

The electronic databases of PubMed, Embase, and Web of Science databases were searched from inception to February 22, 2021 with a strategy of combined terms including (1) “frailty” OR “frail;” and (2) “percutaneous coronary intervention” OR “stent” OR “angioplasty” OR “revascularization” OR “reperfusion” OR PCI. Only studies reported in English were considered. References of related articles or reviews were also analyzed. The full search term for PubMed database was based on keywords as [(“frailty” OR “frail”) AND (“coronary artery disease” OR “angina” OR “myocardial infarction” OR “acute coronary syndrome” OR “percutaneous coronary intervention” OR “major adverse cardiovascular events” OR “CAD” OR “STEMI” OR “NSTEMI” OR “ACS” OR “AMI” OR “PCI”)].

### Study Identification

Studies fulfilled these criteria were used: (1) cohort studies published as full-length papers; (2) included adult patients with CAD; (3) frailty was evaluated during the index hospitalization for PCI and considered as exposure; (4) compared the incidence of all-cause mortality and/or major adverse cardiovascular events (MACEs) between patients with and without frailty during follow-up; and (4) reported risk ratios (RRs) for the above associations after adjusting for multiple confounding factors (at least for age and sex). Methods for the assessment of frailty were in accordance with those applied in the original articles. We defined MACEs as a composite outcome of all-cause death, non-fatal myocardial infarction (MI), non-fatal stroke, repeated coronary revascularization, and cardiac readmission. Reviews, preclinical studies, cross-sectional studies, and irrelevant studies were not included.

### Data Extracting and Quality Evaluation

Two authors implemented database search, data extraction, and study quality assessment separately. If disagreements occurred, they were discussed with the corresponding author. These data were recorded: (1) author, country, and study design characteristics; (2) characteristics of the patients, including the diagnosis, number of participants included, mean age, and sex; (3) methods for the evaluation of frailty and number of patients with frailty at baseline; (4) PCI type and follow-up durations; (5) outcomes reported; and (6) potential confounding factors adjusted in the multivariate analyses for the association. The Newcastle-Ottawa Scale (31) was used for study quality evaluation. This scale is rated from 1 to 9 stars and reflected the quality of the study by aspects of participant selection, comparability between groups, and outcome validation.

### Statistical Analyses

RRs and the corresponding 95% confidence intervals (CIs) were extracted for every included study. Then, standard errors (SEs) of RRs were estimated from the 95% CIs or *P*-values. For normalization of their distribution, HRs were logarithmically transformed (30) and combined. Heterogeneity within the included cohort studies was tested via Cochrane's *Q*-test, as well as the estimation of *I*^2^ statistic (32). An *I*^2^ > 50% suggests significant level of heterogeneity. A random-effects model was chosen to combine the RRs by incorporating the potential heterogeneity within studies (30). Sensitivity analyses by sequentially excluding either of the included studies were conducted to clarify the influence of a certain study on the overall results (33). Predefined subgroup analyses according to study design, follow-up duration, and type of PCI were also performed. Funnel plots were constructed, and were used for the assessment of publication bias (34). Visually asymmetrical funnel plots implied potential publication bias, which could be further validated by the Egger's regression asymmetry test. If high risk of publication bias was suggested, a “trim-and-fill” analysis was used for further evaluation, which estimates the influence of possible studies with negative findings on the meta-analysis outcome (30). The RevMan (Version 5.1; Cochrane Collaboration, Oxford, UK) and STATA software were involved for statistical analyses.

## Results

### Database Search

**Figure 1:**
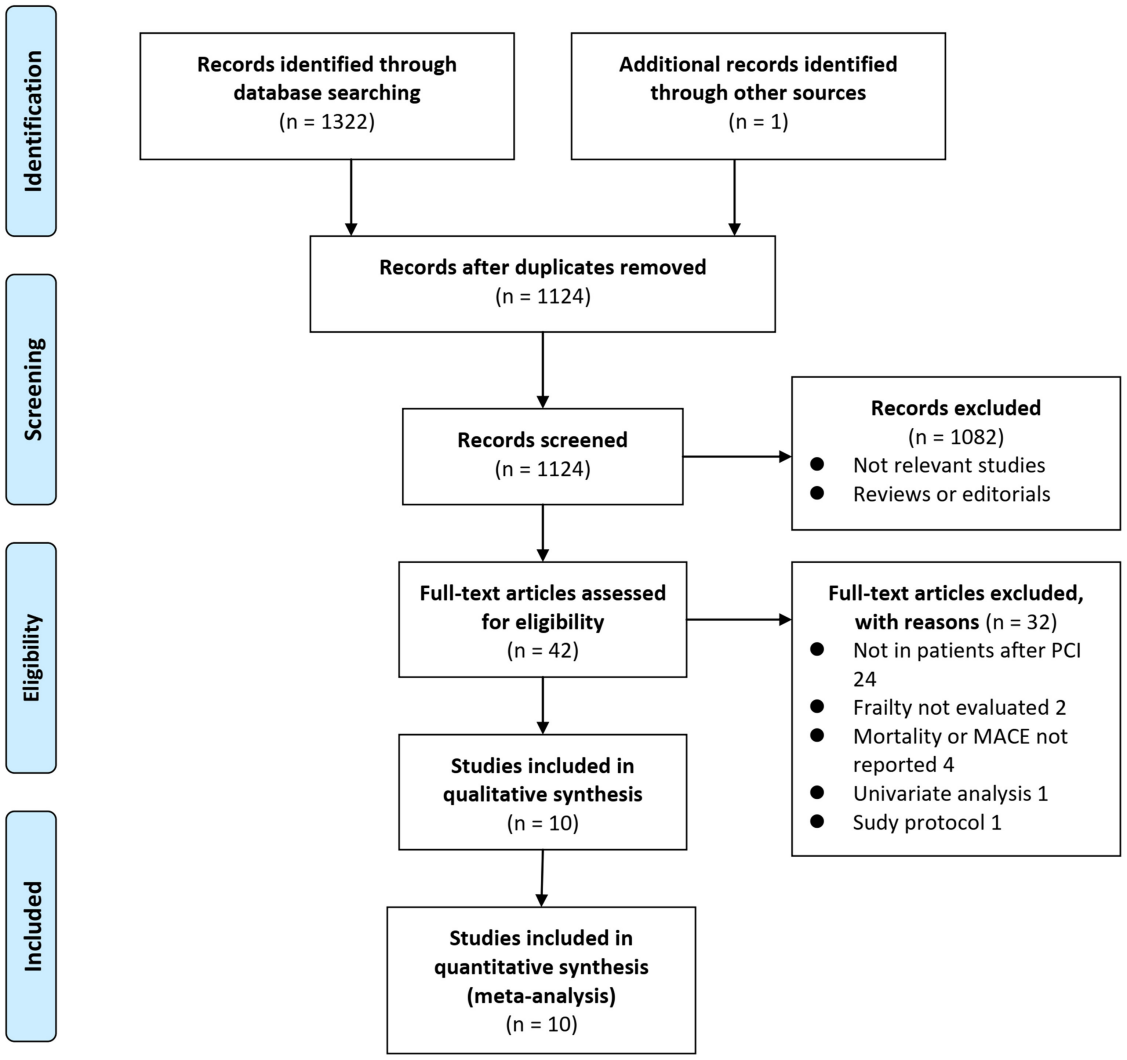
Scheme of study inclusion.

Details of the database search are shown in Figure 1. The first-step database search retrieved 1,124 articles after duplicated studies were excluded. Among them, 1,082 studies were further excluded because they were not related to the purpose of the meta-analysis based on titles and abstracts. Then, for the remaining 42 studies evaluated by full text reading, 32 were not included for the reasons presented in Figure 1, which resulted in ten cohort studies finally analyzed in the meta-analysis (15–24).

### Study Characteristics

Characteristics of each study of the meta-analysis are shown in Table 1. Overall, ten cohort studies with 7,449,001 patients were considered to be eligible for the meta-analysis (15–24), which were performed in the United States (15, 19), United Kingdom (16, 17, 24), Spain (18), Japan (21, 22), and Indonesia (23), respectively. Five of them were prospective (15–18, 23), and the rest were retrospective (19–22, 24). Three studies included patients with unselected CAD (15, 16, 24), the others included patients with stable CAD (23), non-ST segment elevation acute coronary syndrome (NSTE-ACS) (17), and ST-elevation myocardial infarction (18–22), respectively. The mean ages of the included patients varied between 62 and 85 years, with proportions of males varying from 46 to 75%. Multiple tools were used for the evaluation of frailty among the included studies, including the Fried Frailty Criteria (15, 17), the Canadian Study of Health and Aging Clinical Frailty Scale (16, 22), validated frailty phenotype criteria (23), FRAIL scale (18), Claims-based Frailty Index (19), Safety Management Programme Score (20), Hospital Frailty Risk Score (24), and modified KATZ index (21). A total of 16,183 patients were considered with frailty at baseline. All the patients included in these studies received primary or elective PCI procedures. The follow-up durations varied from within hospitalization to 35 months after PCI. Incidence of all-cause mortality was reported in eight studies (15, 16, 18–22, 24), and incidence of MACEs was reported in five studies (15, 17, 20, 23, 24). Age, sex, body mass index, risk factors for CAD, comorbidities, and coronary lesion features were adjusted to a varying degree when the associations between frailty and outcomes after PCI were reported. The quality of these studies was good, evidenced by six to nine points of NOS scores (Table 2).

**Table 1:** Characteristic of the included studies.

**Study**	**Country**	**Design**	**Patient characteristics**	**Sample size**	**Mean age (years)**	**Male (%)**	**Frailty evaluation**	**No. of patients with frailty**	**PCI type**	**Follow-up duration (months)**	**Outcomes**	**Variables adjusted**
Singh et al. (15)	USA	PC	CAD patients ≥ 65 years who underwent PCI	629	69.0	74.3	Using the Fried Frailty Criteria during index hospitalization	117	Primary or elective	35	All-cause mortality and MACE	Age, sex, Mayo Clinic Risk Score, Charlson Comorbidity Index, and Short-form-36
Murali-Krishnan et al. (16)	UK	PC	CAD patients who underwent PCI	746	62.2	70.1	Using the Canadian Study of Health and Aging Clinical Frailty Scale during hospitalization	81	Primary or elective	12	All-cause mortality	Age, sex, hemodynamically instability, CHF, DM, COPD, renal failure, and TIA/stroke
Hamonangan et al. (23)	Indonesia	PC	Stable CAD patients≥ 60 years who underwent PCI	100	70.0	69.0	Using frailty phenotype criteria during hospitalization	61	Elective	1	MACE	Age, sex and comorbidities
Calvo et al. (18)	Spain	PC	STEMI patients ≥ 75 years who underwent primary PCI	259	82.6	57.9	Using FRAIL scale during hospitalization	51	Primary	In-hospitalization	All-cause mortality	Age, sex, LVEF, number of vessels diseased, and Barthel index
Batty et al. (17)	UK	PC	NSTEACS patients ≥ 75 years who underwent PCI	280	81.0	60.0	Using Fried Frailty Index during hospitalization	77	Elective	12	MACE	Age, sex, SBP, Killip Class, history of PVD, and BMS use
Damluji et al. (19)	USA	RC	STEMI patients ≥ 75 years who underwent primary PCI	140,089	80.9	51.0	Using Claims-based Frailty Index during hospitalization	13,855	Primary	In-hospitalization	All-cause mortality	Age, sex, and comorbidities
Hermans et al. (20)	The Netherlands	RC	STEMI patients ≥ 70 years who underwent primary PCI	206	79.0	58.0	Using Safety Management Programme Score during hospitalization	57	Primary	1	All-cause mortality and MACE	Age, sex, CAD risk factors, comorbidities, and treatments
Yoshioka et al. (22)	Japan	RC	STEMI patients ≥ 80 years who underwent primary PCI	273	84.6	46.2	Using the Canadian Study of Health and Aging Clinical Frailty Scale at admission	34	Primary	24	All-cause mortality	Age, sex, CAD risk factors, comorbidities, and coronary lesion features
Kwok et al. (24)	UK	RC	CAD patients who underwent PCI	730,6007	66.1	65.3	Using a validated Hospital Frailty Risk Score during hospitalization	1,836	Primary or elective	In-hospitalization	All-cause mortality and MACE	Age, sex, Charlson Comorbidity Index, and coronary lesion features
Seguchi et al. (21)	Japan	RC	STEMI patients ≥ 80 years who underwent primary PCI	412	84.5	60.0	Using the modified KATZ index during hospitalization	14	Primary	In-hospitalization	All-cause mortality	Age, sex, Killip Class, hemoglobin, comorbidities, and treatments

*PCI, percutaneous coronary intervention; PC, prospective cohort; RC, retrospective cohort; CAD, coronary artery disease; STEMI, ST-segment elevation myocardial infarction; NSTE-ACS, non-ST-segment elevation acute coronary syndrome; MACE, major adverse cardiovascular events; CHF, congestive heart failure; DM, diabetes mellitus; COPD, chronic obstructive pulmonary disease; TIA, transient ischemia attack; LVEF, left ventricular ejection fraction; SBP, systolic blood pressure; PVD, peripheral vascular disease; BMS, bare-metal stent*.

**Table 2:** Study quality evaluation via the newcastle-ottawa scale.

**Study**	**Representativeness of the exposed cohort**	**Selection of the non-exposed cohort**	**Ascertainment of exposure**	**Outcome not present at baseline**	**Control for age and sex**	**Control for other confounding factors**	**Assessment of outcome**	**Enough long follow-up duration**	**Adequacy of follow-up of cohorts**	**Total**
Singh et al. (15)	1	1	1	1	1	0	1	1	1	8
Murali-Krishnan et al. (16)	1	1	1	1	1	1	1	1	1	9
Hamonangan et al. (23)	0	1	1	1	1	0	1	0	1	6
Calvo et al. (18)	0	1	1	1	1	1	1	0	1	7
Batty et al. (17)	0	1	1	1	1	1	1	1	1	8
Damluji et al. (19)	0	1	1	1	1	0	1	0	1	6
Hermans et al. (20)	0	1	1	1	1	1	1	0	1	7
Yoshioka et al. (22)	0	1	1	1	1	1	1	1	1	8
Kwok et al. (24)	1	1	1	1	1	1	1	0	1	8
Seguchi et al. (21)	0	1	1	1	1	1	1	0	1	7

### Association Between Frailty and All-Cause Mortality After PCI

**Figure 2:**
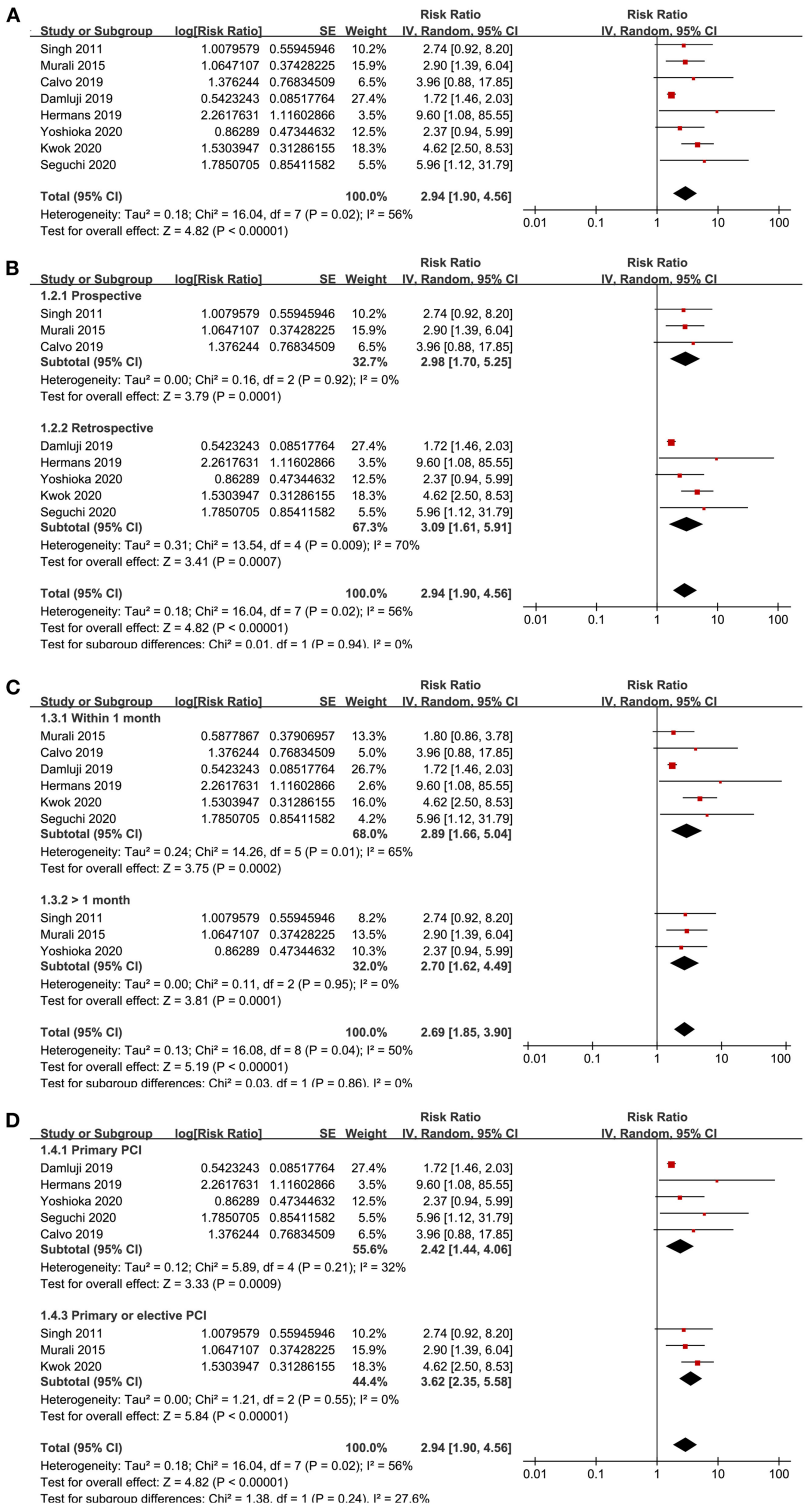
Forest plots for the meta-analysis concerning the association between frailty and risk of all-cause mortality after PCI; **(A)**, Overall meta-analysis; **(B)**, Subgroup analysis according to study design; **(C)**, Subgroup analysis according to follow-up duration; and **(D)**, Subgroup analysis according to the type of PCI.

Eight studies (15, 16, 18–22, 24) reported the outcome of all-cause mortality after PCI. Moderate heterogeneity was detected among the included studies (P for Cochrane's *Q*-test = 0.02, *I*^2^ = 56%). Pooled results with a random-effect model showed that frailty was independently associated with a higher incidence of all-cause mortality (adjusted RR = 2.94, 95% CI: 1.90–4.56, *P* < 0.001; Figure 2A). Sensitivity analyses by excluding one study at a time showed similar results (RR: 2.33–3.54, *P* all < 0.05). Sensitivity analysis only including studies with elderly patients (15, 18–22) showed consistent results (adjusted RR = 2.27, 95% CI: 1.51–3.41, *P* < 0.001), and the heterogeneity was substantially reduced (P for Cochrane's *Q*-test = 0.26, *I*^2^ = 23%). Subgroup analyses showed that the association between frailty and increased risk of all-cause mortality in patients with CAD after PCI was not significantly affected by characteristics of study design (prospective or retrospective), follow-up duration (within or more than 1 month), or type of PCI (primary or elective; *P*-values for subgroup analyses all >0.05; Figures 2B–D).

### Association Between Frailty and MACEs After PCI

**Figure 3:**
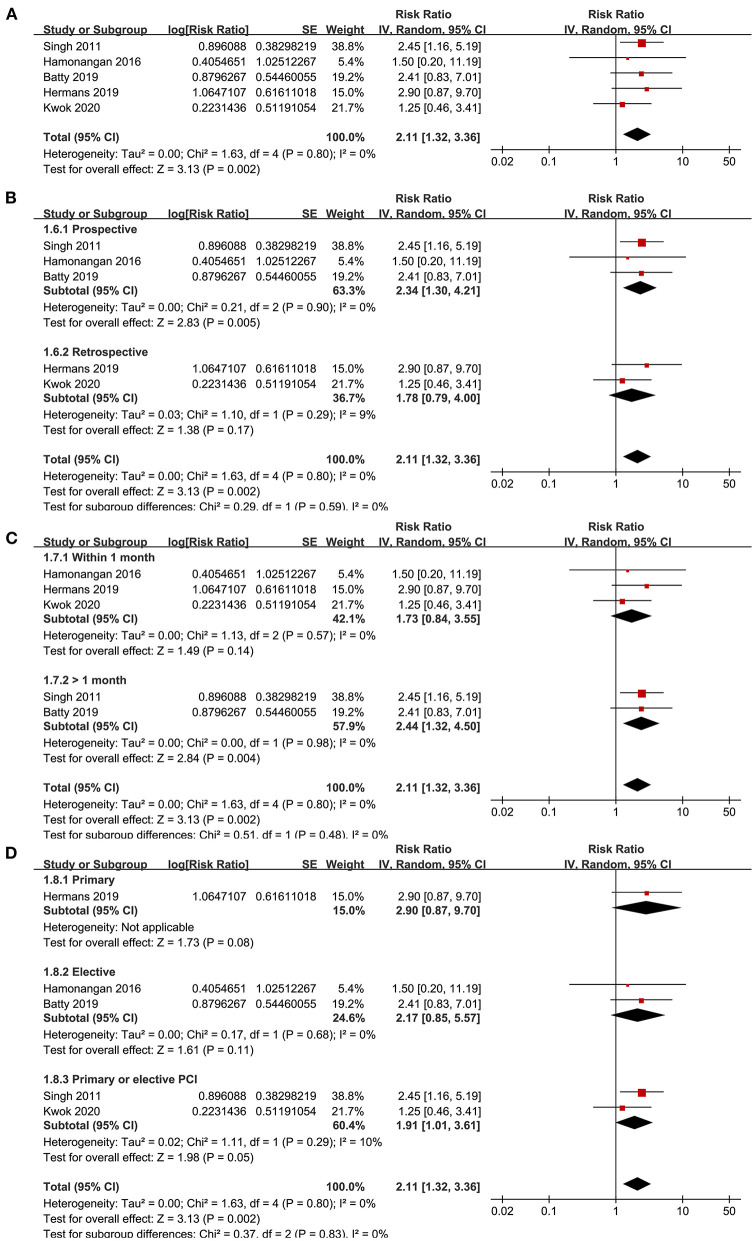
Forest plots for the meta-analysis concerning the association between frailty and risk of MACEs after PCI; **(A)**, Overall meta-analysis; **(B)**, Subgroup analysis according to study design; **(C)**, Subgroup analysis according to follow-up duration; and **(D)**, Subgroup analysis according to the type of PCI.

Five studies (15, 17, 20, 23, 24) reported the outcome of MACEs. No significant heterogeneity was detected (*P* for Cochrane's *Q*-test = 0.80, *I*^2^ = 0%). Pooled results showed that frailty was independently associated with a higher incidence of MACEs (adjusted RR = 2.11, 95% CI: 1.32–3.66, *P* = 0.002; Figure 3A). Sensitivity analyses by excluding one study at a time showed similar results (RR: 1.92–2.44, *P* all < 0.05). Sensitivity analysis limited to studies with elderly patients (15, 17, 20, 23) also showed consistent results (adjusted RR = 2.44, 95% CI: 1.44–4.13, *P* = 0.001; *I*^2^ = 0%). Subgroup analyses also showed that characteristics of study design, follow-up duration, or type of PCI did not significantly affect the association (*P*-values for subgroup analyses all >0.05; Figures 3B–D).

### Publication Bias

**Figure 4:**
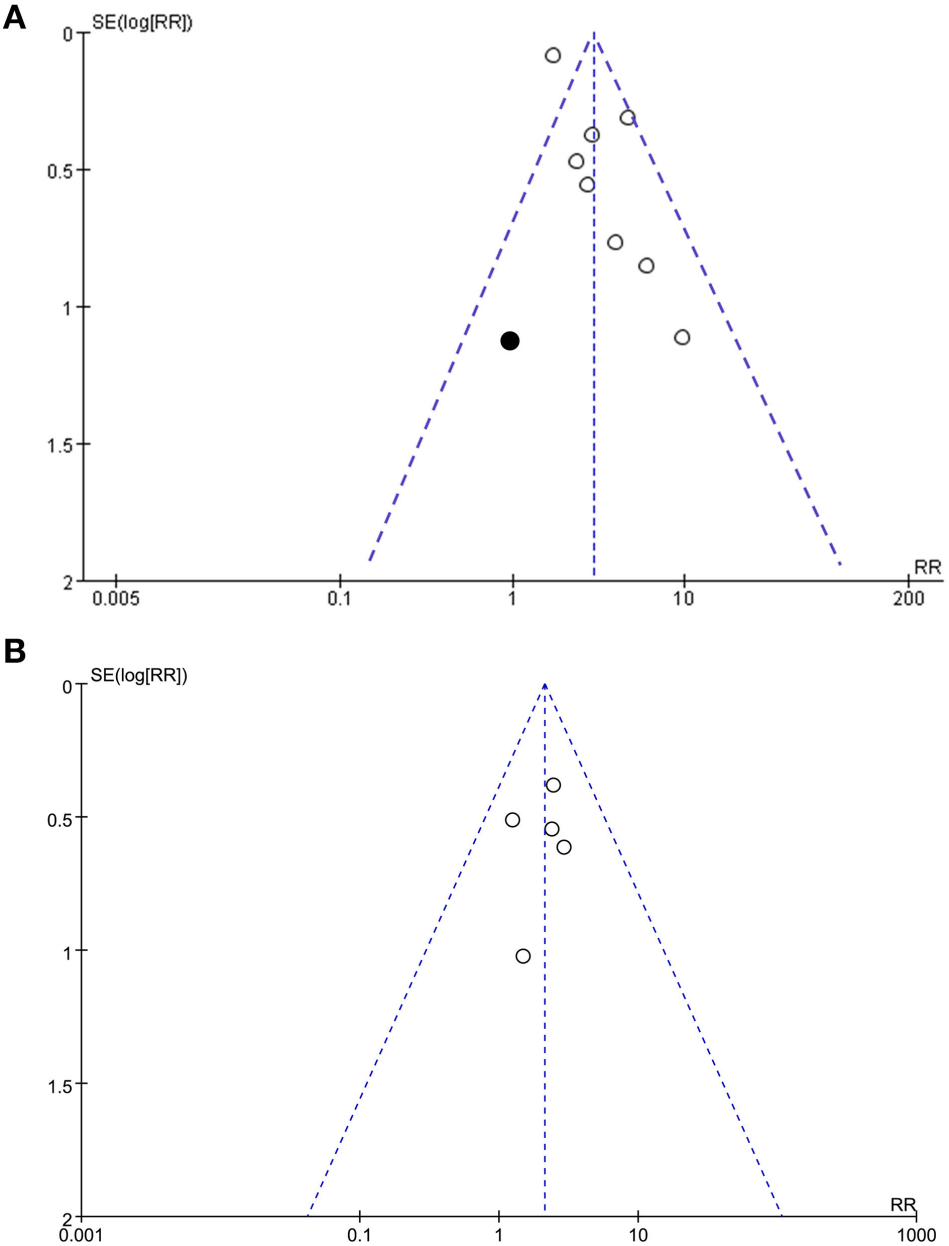
Funnel plots for the meta-analyses; **(A)**, Funnel plots with trim-and-fill analysis for the meta-analysis concerning the association between frailty and risk of all-cause mortality after PCI (black square indicates the hypothesize study to achieve the symmetry of the funnel plots); and **(B)**, Funnel plots for the meta-analysis concerning the association between frailty and risk of MACEs after PCI.

Funnel plots representing the meta-analyses for the associations between frailty and all-cause mortality after PCI were shown in Figure 4A. The plots for the outcome of all-cause mortality were asymmetrical based on visual inspection, raising the possible publication bias (Figure 4A). Egger's regression test also demonstrated potential risk of publication bias (*P* = 0.048). We therefore performed a trim-and-fill analysis. As shown in Figure 4A, incorporating a hypothesized study achieved symmetry of the funnel plots, and the results of the meta-analysis remained significant after including the study (adjusted RR = 2.80, 95% CI: 1.83–4.27, *P* < 0.001; *I*^2^ = 52%). Funnel plots representing the meta-analyses for the associations between frailty and MACEs after PCI were shown in Figure 4B. These plots were symmetrical judged by visual inspection, reflecting low possibility of publication bias. Egger's regression test was not performed because only five datasets were analyzed for this outcome.

## Discussion

In this meta-analysis of cohort studies, we found that frailty was independently associated with higher incidences of morality and MACEs in patients with CAD after PCI. Sensitivity analyses showed that the significance of the results was not affected by omitting of either of the included studies. Besides, sensitivity analyses limited to studies including elderly patients with CAD showed consistent results. Moreover, results of subgroup analyses showed that the association between frailty and poor prognosis after PCI was not significantly affected by study characteristics such as study design, follow-up duration, and type of PCI. Although risk of publication bias was noticed for the meta-analysis of the association between frailty and all-cause mortality after PCI, results of trim-and-fill analysis by incorporating the imputed study with negative result showed consistent results. Taken together, results of these findings indicated that frailty may be an independent risk factor for poor prognosis in patients with CAD who were treated with PCI.

A previous meta-analysis published in 2017 also showed that frailty may be associated with higher mortality risk for CAD patients after PCI (25). However, several weaknesses regarding the methodology of the meta-analysis have been noticed, which may affect the interpretation of the results. Besides of studies published as full-length articles that underwent peer-review, this meta-analysis has also included studies published in conference abstracts, which may introduce bias to the results. Moreover, this meta-analysis included two studies that not all of the included patients were treated with PCI (26, 27). In addition, one study with frailty measured via gait speed as a continuous variable was also introduced into the meta-analysis, which may confound the results of the meta-analysis (28). Compared to this study, our meta-analysis has several strengths. Firstly, only studies published as full-length articles were included. Secondly, all of the studies included patients with CAD who were treated with PCI. Thirdly, comparisons for the incidence of adverse clinical outcomes were directly made between patients with and without frailty. Moreover, besides all-cause mortality, outcome of MACEs was also evaluated. In addition, only studies with multivariate analyses were considered, which therefore could indicate a potentially independent association between frailty and poor outcomes after PCI. Finally, multiple sensitivity and subgroup analyses were performed, which showed consistent results in elderly patients, in studies with different design, follow-up durations, and types of PCI.

The potential mechanisms underlying the association between frailty and poor outcomes after PCI remain not fully understood. It has been shown that frail patients may have longer recovery time after invasive procedures (16), suggesting these patients may suffer from more post-procedure complications (23). Moreover, frailty has been associated with endothelial dysfunction (35) and activated inflammatory response (36), two key molecular mechanisms underlying the adverse events after PCI, such as in-stent restenosis (37, 38). In addition, in a recent study in elderly Chinese CAD patients after PCI, frailty has been related to high on-aspirin platelet response and high on-clopidogrel platelet response among, a validated independent risk factor of thrombotic events after PCI (39). Further studies are warranted to evaluate the exact mechanisms involved in the association between frailty and poor prognosis after PCI.

It has to be mentioned that although we found that patients with frailty may have increased risk of mortality and MACEs after PCI, it does not mean that PCI should be avoided in patients with frailty. In fact, it was shown that STEMI patients with frailty had reduced hospital mortality after PCI as compared to those who received conservative treatments only (19). Since we have shown that frailty may be an independent risk factor for poor prognosis after PCI, it could be hypothesized that whether strategies alleviating frailty in these patients could provide additional clinical benefits after PCI. Studies are needed for further investigations.

This study also has limitations. Firstly, the meta-analysis was not registered at PROSPERO prospectively, but we followed the predefined protocol during the performance of the study. Secondly, the meta-analysis was not based on data from the study level but not from individual patients, which prevented further analyses on the influence of patient characteristics on the outcome. In addition, significant heterogeneity was detected for the meta-analysis of the association between frailty and mortality after PCI. Although sensitivity analysis limited to studies including elderly patients only significant reduced the heterogeneity (*I*^2^ from 56 to 23%), we could not determine whether other factors contribute to the residual heterogeneity. Moreover, multiple scales were used for measurement of frailty, and we could not determine whether difference among these scales may affect the association between frailty and outcomes after PCI. Finally, possible risk of publication bias was raised in the meta-analysis regarding the association between frailty and poor prognosis after PCI. However, further trim-and-fill analysis suggested that the potential publication bias was not likely to affect the finding.

In conclusion, this updated meta-analysis of cohort studies suggested that frailty may be an independent risk factor of poor prognosis for patients with CAD after PCI. Future studies are needed to determine the optimal measurement tool for frailty for patients undergoing PCI, and to evaluate whether strategies to attenuate frailty could provide additional clinical benefits in these patients.

## Data Availability Statement

The original contributions presented in the study are included in the article/supplementary material, further inquiries can be directed to the corresponding authors.

## Author Contributions

PW and JT designed the study. PW and SZ performed literature search, study quality evaluation, and data extraction. PW and KZ performed statistical analyses. PW, SZ, and KZ interpreted the results. PW drafted the manuscript. All authors contributed to the article and approved the submitted version.

## Conflict of Interest

The authors declare that the research was conducted in the absence of any commercial or financial relationships that could be construed as a potential conflict of interest.

## Publisher's Note

All claims expressed in this article are solely those of the authors and do not necessarily represent those of their affiliated organizations, or those of the publisher, the editors and the reviewers. Any product that may be evaluated in this article, or claim that may be made by its manufacturer, is not guaranteed or endorsed by the publisher.
